# ChromaClade: combined visualisation of phylogenetic and sequence data

**DOI:** 10.1186/s12862-019-1518-9

**Published:** 2019-10-15

**Authors:** Christopher Monit, Richard A. Goldstein, Greg J. Towers

**Affiliations:** Division of Infection and Immunity, University College London, London, WC1E 6BT UK

**Keywords:** Phylogenetics, Protein evolution, Visualisation

## Abstract

**Background:**

Studying site-specific amino acid frequencies by eye can reveal biologically significant variability and lineage-specific adaptation. This so-called ‘sequence gazing’ often informs bioinformatics and experimental research. But it is important to also account for the underlying phylogeny, since similarities may be due to common descent rather than selection pressure, and because it is important to distinguish between founder effects and convergent evolution. We set out to combine phylogenetic and sequence data to produce evolutionarily insightful visualisations.

**Results:**

We present ChromaClade, a convenient tool with a graphical user-interface that works in concert with popular tree viewers to produce colour-annotated phylogenies highlighting residues found in each taxon and at each site in a sequence alignment. Colouring branches according to residues found at descendent tips also quickly identifies lineage-specific residues and those internal branches where key substitutions have occurred. We demonstrate applications of ChromaClade to human immunodeficiency virus and influenza A virus datasets, illustrating cases of conservative, adaptive and convergent evolution.

**Conclusions:**

We find this to be a powerful approach for visualising site-wise residue distributions and detecting evolutionary patterns, especially in large datasets. ChromaClade is available for Windows, macOS and Unix or Linux; program executables and source code are available at github.com/chrismonit/chroma_clade.

## Background

Visually inspecting molecular sequence data – so called ‘sequence gazing’ – can be extremely insightful. Sites that are evolutionarily conserved may be crucial for a protein’s structure and function, while variation may indicate divergent selective constraints in separate groups. For molecular biologists these observations can inspire hypotheses to be tested by point mutation experiments that examine the functional effect of the differences, while for bioinformaticians they can motivate more formal computational analysis. The approach is vastly improved by taking account of the organisms’ phylogenetic relationships, firstly because some sequences will be more similar due to common descent rather than evolutionary constraint and secondly because this helps identify evolutionary trends, such as characteristics gained or lost in particular clades. At present there is no convenient, automated way to visualise phylogenetic and sequence data simultaneously.

## Implementation

ChromaClade annotates and colours taxon names in phylogenetic trees according to the residues found in the corresponding sequence alignment. For each site in an alignment, ChromaClade annotates taxon names with residue letter codes and a residue-specific hexadecimal red/green/blue colour code that can be recognised by popular tree viewers, such as FigTree [1] or Archaeopteryx [2]. The annotated trees, whose topologies are identical to the original, are saved to a single file to be loaded into the tree viewer, meaning the data for each site can be inspected by simply looking through the set of coloured trees in the viewer.

Residue-specific colouring can also be applied to branches whose descendent taxa have the same residue. This helps classify lineages by their unique amino acids and illustrates how deep within the phylogenetic history substitutions have arisen.

Written in Python, ChromaClade is suitable for all major operating systems and has graphical and command line interfaces available; convenient application bundles are available for Windows and macOS. ChromaClade makes use of the Biopython and Biopython.Phylo libraries [3].

## Results

We present example applications of ChromaClade using human immunodeficiency virus type 1 (HIV-1) and influenza A virus (IAV) datasets where colour-annotating trees highlights differences already known to be biologically significant, illustrating how the approach can be used prospectively.

**Fig. 1:**
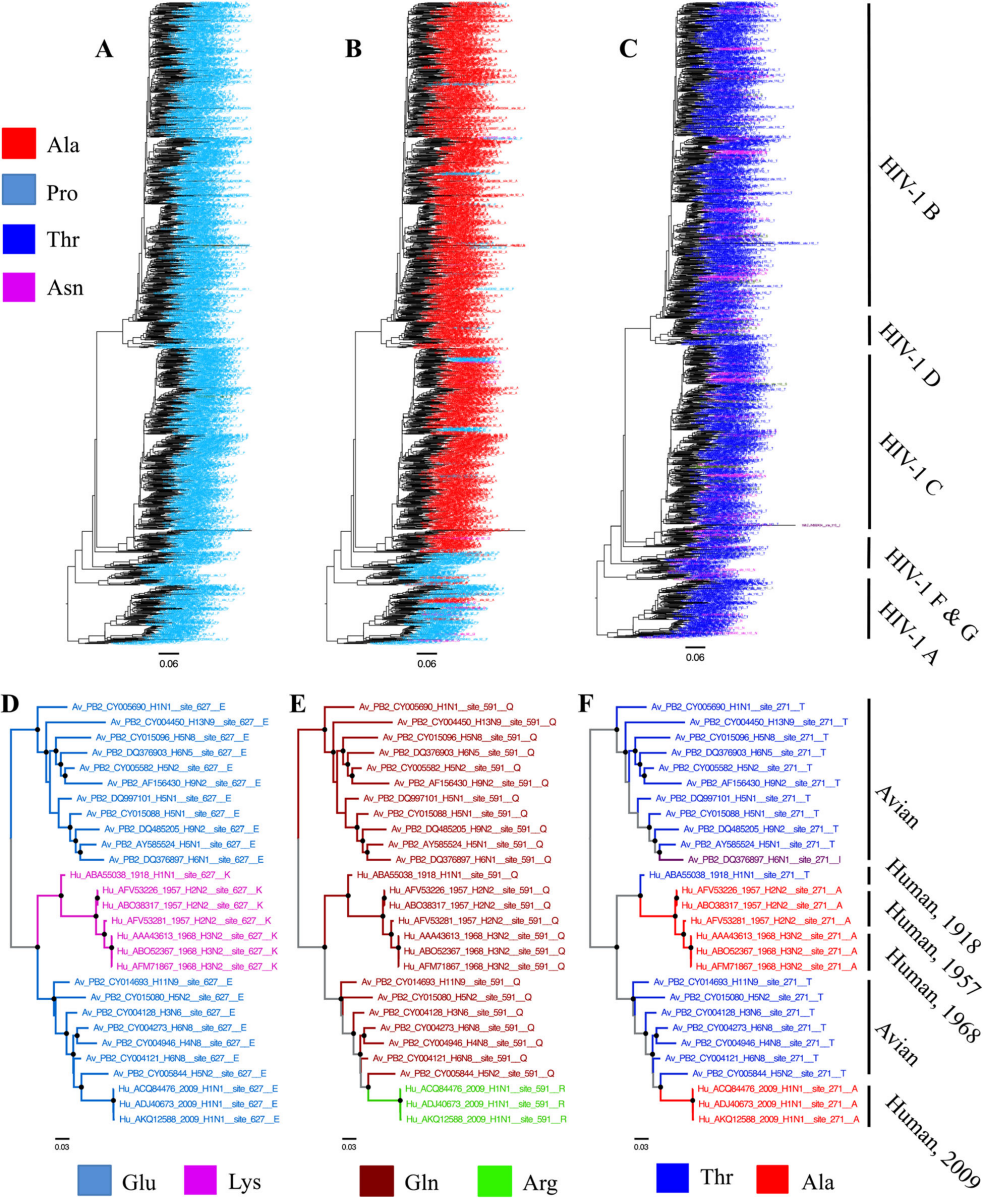
ChromaClade example applications. **a-c** A dataset of 1331 HIV-1 group M capsid sequences containing representatives from all subtypes was downloaded from the Los Alamos HIV-1 sequence database [4] and aligned manually. The phylogeny was estimated from the nucleotide sequences using RAxML 8 [5] with substitution model GTR + Gamma and rooted using HIV-1 group O sequences as an outgroup (not shown). ChromaClade was used to annotate taxon labels with residues found at capsid protein sites. **a** Site 1, proline is entirely conserved; **b** site 92, alanine is mostly conserved in subtypes, **B**, **C** and **D**, while proline is mostly conserved in the remaining subtypes; **c**, site 110, the wildtype threonine is found in most sequences, while the asparagine escape mutant has arisen multiple times independently. Prominent subtypes are indicated, right. **d-f** A phylogeny was estimated as above for an aligned set of avian and pandemic human influenza virus PB2 gene sequences downloaded from the influenza virus resource [6] and mid-point rooted; the sampling years of the human pandemic sequences are shown, right. Black circles indicate clades found in at least 700 of 1000 bootstrap replicates. ChromaClade was used to colour-annotate the taxon labels and branches according to residues found at sites 627 (**d**), 591 (**e**) and 271 (**f**); branches where the ancestral state is unclear are coloured grey. These annotated trees were visualised using FigTree [1]

Large sequence datasets are increasingly available and our approach gives an immediate impression of the variability in a dataset while showing the important phylogenetic context. We applied ChromaClade to a dataset of > 1300 published pandemic HIV-1 capsid gene sequences including all virus subtypes, using a maximum likelihood phylogeny. The annotated trees illustrate conservation (Fig. 1a) and striking lineage-specific differences that suggest possible adaptation to divergent selective constraints across viral subtypes (Fig. 1b). Moreover, it is possible to observe adaptive evolution at sites such as 110, where the T110 N substitution is associated with escape from the host’s cellular immune system but often reverts upon transmission to an uninfected individual (Fig. 1c). Combining phylogenetic and sequence data shows T110 N has arisen many times independently, consistent with the model of escape followed by reversion [7].

Colour-annotating trees reveals those sites where genotypic divergence may underpin experimentally observed phenotypic differences. Moreover, branches can be coloured according to residues observed in descendant taxa, showing the point in evolutionary history when significant substitutions occurred. We applied ChromaClade to a dataset of published sequences from the *PB2* gene that codes for part of the viral polymerase complex in IAV, including human IAV pandemic isolates and avian lineage isolates from which these ultimately descend. At site 627 in PB2, substitution of glutamic acid found in avian IAV to lysine confers enhanced replication in mammalian cells and has been associated with each human IAV pandemic of the twentieth century [8]. We found residue and branch annotation of the *PB2* phylogeny clearly marks E627K as a lineage-specific genotype among earlier pandemic isolates (Fig. 1d). Isolates from the 2009 H1N1 IAV pandemic possessed the avian E627, but interestingly underwent compensatory substitutions at nearby sites which conferred a fitness increase in human cells similar to E627K [9]; again, these substitutions are clearly visible from the annotated trees (Fig. 1e). This illustrates that comparing annotated trees from multiple sites allows the user to spot compensatory substitutions or other potential evolutionary dependence between sites.

Further PB2 substitutions have been linked to higher replication efficiency in mammalian cells following transmission from birds, for example T271A [10]. Studying annotated trees for this site revealed striking convergent evolution, as these substitutions have arisen independently in separate human IAV PB2 lineages (Fig. 1f). This is only apparent if phylogenetic relationships and sequence data are visualised together.

## Conclusions

We have found colour-annotating phylogenies to be an extremely powerful way to inspect sequence data and form testable experimental hypotheses in our own research [11]. While colour-annotations can be produced manually using a tree viewer for small trees and a handful of alignment sites, ChromaClade makes this possible for datasets containing hundreds of taxa and alignment sites. We recommend ChromaClade as a useful exploratory tool for linking phenotype to genotype when studying any group of related organisms.

## Availability and requirements

Project name: ChromaClade.

Project home page: https://github.com/chrismonit/chroma_clade

Operating system(s): Platform independent.

Programming language: Python.

Other requirements: Command line version requires Python 3 and Python module Biopython; Linux/Unix graphical version also requires Python module Pillow.

License: Apache 2.0.

Any restrictions to use by non-academics: None.

## Data Availability

ChromaClade Python source code and application files for Windows and macOS are available at https://github.com/chrismonit/chroma_clade under the Apache License 2.0. Data analysed are publicly accessible from the Los Alamos HIV Sequence Database (https://www.hiv.lanl.gov/content/sequence/HIV/mainpage.html) and the Influenza Virus Resource at the National Center for Biotechnology Information (https://www.ncbi.nlm.nih.gov/genomes/FLU/Database/nph-select.cgi).

## Abbreviations

HIV-1: human immunodeficiency virus type 1
IAV: Influenza A virus

## References

[1] Rambaut A (2006). FigTree, version 1.4.4.

[2] Zmasek CM, Eddy SR (2001). ATV: display and manipulation of annotated phylogenetic trees. Bioinformatics.

[3] Talevich E, Invergo BM, Cock PJ, Chapman BA (2012). Bio.Phylo: A unified toolkit for processing, analyzing and visualizing phylogenetic trees in Biopython. BMC Bioinformatics.

[4] HIV Sequence Database. Los Alamos National Laboratory, USA. 2013. http://www.hiv.lanl.gov/. Accessed 10 Jan 2018.

[5] Stamatakis A (2014). RAxML version 8: a tool for phylogenetic analysis and post-analysis of large phylogenies. Bioinformatics.

[6] Bao Y, Bolotov P, Dernovoy D, Kiryutin B, Zaslavsky L, Tatusova T (2008). The influenza virus resource at the National Center for biotechnology information. J Virol.

[7] Brockman MA, Schneidewind A, Lahaie M, Schmidt A, Miura T, DeSouza I (2007). Escape and compensation from early HLA-B57-mediated cytotoxic T-lymphocyte pressure on human immunodeficiency virus type 1 gag Alter capsid interactions with Cyclophilin A. J Virol.

[8] Subbarao EK, London W, Murphy BR (1993). A single amino acid in the PB2 gene of influenza a virus is a determinant of host range. J Virol.

[9] Mehle A, Doudna JA (2009). Adaptive strategies of the influenza virus polymerase for replication in humans. Proc Natl Acad Sci.

[10] Bussey KA, Takimoto T, Kim B, Desmet EA, Bousse TL (2010). PB2 residue 271 plays a key role in enhanced polymerase activity of influenza A viruses in mammalian host cells. J Virol.

[11] Monit C, Morris ER, Ruis C, Szafran B, Thiltgen G, Tsai M-HC (2019). Positive selection in dNTPase SAMHD1 throughout mammalian evolution. Proc Natl Acad Sci.

